# Gut Feelings: How Microbiota Might Impact the Development and Course of Anorexia Nervosa

**DOI:** 10.3390/nu12113295

**Published:** 2020-10-28

**Authors:** Jochen Seitz, Brigitte Dahmen, Lara Keller, Beate Herpertz-Dahlmann

**Affiliations:** Department of Child and Adolescent Psychiatry, Psychotherapy and Psychosomatics, RWTH University Hospital, Neuenhofer Weg 21, 52074 Aachen, Germany; bdahmen@ukaachen.de (B.D.); lkeller@ukaachen.de (L.K.); bherpertz@ukaachen.de (B.H.-D.)

**Keywords:** anorexia nervosa, gut microbiota, diet, nutrition, gut brain interaction, Lachnospiraceae, prediction, autoimmune, inflammation, gut permeability

## Abstract

Anorexia nervosa (AN) can probably be regarded as a “model” for studying the interaction of nutrition with the gut–brain axis, which has drawn increased attention from researchers and clinicians alike. The gut microbiota influences somatic effects, such as energy extraction from food and body weight gain, as well as appetite, gut permeability, inflammation and complex psychological behaviors, such as depression or anxiety, all of which play important roles in AN. As nutrition is one of the main factors that influence the gut microbiota, nutritional restriction and selective eating in AN are likely influencing factors; however, nutritional rehabilitation therapy is surprisingly understudied. Here, we review the general mechanisms of the interactions between nutrition, the gut microbiota and the host that may be relevant to AN, paying special attention to the gut–brain axis, and we present the first specific findings in patients with AN and corresponding animal models. In particular, nutritional interventions, including food selection, supplements, and pre-, pro- and synbiotics that have the potential to influence the gut microbiota, are important research targets to potentially support future AN therapy.

## 1. Introduction

According to the DSM-5, anorexia nervosa (AN) is classified as a combination of low body weight, distorted body image and fear of gaining weight [1,2]. AN leads to severe somatic and endocrine consequences, including amenorrhea, low leptin and triiodothyronine levels, and increased ghrelin and cortisol levels. The etiology of AN is not well understood; approximately 60% of the variance is genetic or epigenetic in origin, and psychosocial and individual factors also play important roles [3]. Due to its recently identified genetic associations with low body mass index, low incidence of type 2 diabetes, obesity, low fasting insulin and high HDL cholesterol levels, AN is now regarded as being of metabo-psychiatric origin [4]. AN has an incidence rate of 100–200/100,000 person-years in adolescent females, similar to that of type 1 diabetes [1,2], and a lifetime prevalence of 1–4% in Europe; it is thus one of the most common chronic illnesses in this age group [3]. In Germany and the UK, the numbers of hospital admissions for AN, especially in childhood and adolescence, have substantially risen in recent years [5,6], and the relapse rates remain high due to its often chronic course, culminating in the highest mortality rate of all psychiatric disorders [7]. The emotional burden for patients and their families and the socioeconomic costs to society due to treatment, care and missed work hours for patients with ANare approximately in the same range as those for depression and schizophrenia patients [8,9]. Multimodal treatment includes weight rehabilitation and psychotherapy but is far from sufficient. The underlying pathophysiology and factors that contribute to the chronicity of AN remain understudied.

**Figure 1:**
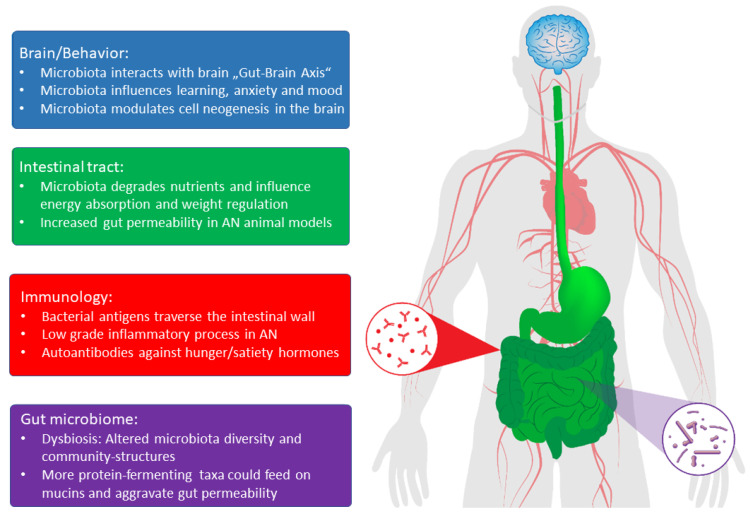
Effects of gut microbial products on brain functions and behavior in the intestinal tract and on immunological processes in patients with AN. (Adapted from [30]).

To date, there are only nine studies on the alterations in the microbiota in patients with AN [10,11,12,13,14,15,16,17] and only two case studies of human fecal transplantation [18,19]. These studies suggest that the gut microbiota is altered in patients with AN, while transplantation of patient stool into rodents suggests that these alterations likely play an important role at least in the maintenance of the disease [20] if not in its etiology (see Figure 1). We thus attempt to present these early findings as well as possible general mechanisms in the interplay of gut microbiota and the host to formulate hypotheses. Some of these mechanisms could also explain specific symptoms of AN. However, research on AN significantly lags behind research on the microbiota as they relate to intestinal disorders, obesity or psychiatric illnesses, such as depression, as this research has already delineated some of these mechanisms. With this article, we thus hope to inspire similar research in the field of eating disorders and AN.

The gut microbiota and its interaction with the host are currently drawing increased attention from researchers and clinicians with respect to multiple diseases, both somatic and psychiatric [21,22]. There are now over 2000 microbial species known to inhabit the human gut [23], and each individual has a “personal” combination of approximately 500 species with a total weight of over 1 kg [24]. The major influencing factor that affect this combination of species is nutrition, while genetics, current illnesses, medication, stress, exercise and past experiences (such as mode of birth, having been breastfed, past illnesses and lifetime use of antibiotics) also appear to contribute [21,22]. Host–microbe interactions are equally manifold; as human evolution took place in a world full of microbes, symbiotic partnerships provided evolutionary advantages to both the microbes and host [25]. Gut microbes perform many functions, e.g., in the digestive system, gut microbes break down food with enzymes not expressed by humans, allowing for a greater diversity of nutrients to be used and more derivatives to be extracted, including fatty acids or essential vitamins [22]. Thus, the gut microbiota can increase or decrease the amount of energy extracted from the same amount of food, which is important for weight regulation. However, host–microbe interactions also include continuous cross-talk with intestinal cells, which influences gut-wall permeability, inflammatory and immunological processes and the secretion of gastric and bacteria-derived hormones [26]. Increasingly, complex direct and indirect interactions between the microbiota, gut and brain, termed the “microbiota-gut–brain axis”, have become increasingly apparent, and microbiota causally influence complex behaviors, such as learning, stress, depression and anxiety [22,27], all of which also play important roles in AN [3]. The potential mechanisms involve direct vagal nerve signaling, migration of immune cells into the brain, and changes in the secretion of cytokines, antibodies, hormones and nutritional components [21,22]. AN can probably be regarded as a helpful model for studying and learning about host-microbiota interactions, since AN is characterized by different factors, such as altered nutrition, weight, hormonal levels, gastrointestinal function and behaviors and the possibility of modifying these factors within a relatively short amount of time during therapy. Furthermore, re-alimentation is a key factor in AN therapy, but controlled studies on how to refeed, with which nutrients and at what rate, remain scarce [28]. Given the important role of nutrition in the gut microbiota, the specific effects of re-alimentation remain unknown; failing to take into account interactions with gut microbes might actually cause iatrogenic harm [29]. On the other hand, nutritional interventions and pre- or probiotics (food enabling the growth of beneficial bacteria or beneficial bacteria themselves) could positively influence the gut microbiota and are currently becoming interesting research targets to complement current AN treatment. In this review, we first describe the most important general findings with regard to the mechanisms of host-microbiota interactions. Then, we describe the potential role of these interactions in the pathophysiology and clinical course of patients with AN.

## 2. Body Weight

In 2005, Ley et al. [31] discovered that overweight mice have increased Firmicutes and a 50% reduction in Bacteroidetes gut bacteria compared to normal-weight mice. Turnbough et al. [32] showed that these bacteria seem to extract more energy from the same amount of food, e.g., by their capacity to metabolize formerly unused fibers into short-chain fatty acids, such as butyrate, propionate and acetate, which are then used by colonic epithelial cells and for lipogenesis. Germ-free raised mice (GF mice) transplanted with stool from obese patients exhibited more weight gain than mice transplanted with stool from normal-weight patients, despite receiving the same amount of food [33]. This interaction of the gut microbiota with the ingested diet regarding weight gain was further emphasized by Tremaroli et al., who used the stool of patients after bariatric surgery and showed a normalization of previously increased energy extraction by gut bacteria [34]. Conversely, supplementation with *Akkermansia muciniphila* as probiotic bacteria resulted in a moderate 3-kg weight loss within 3 months in obese patients in a randomized controlled study [35]. In contrast, the stool of Malawian children with kwashiorkor was transplanted into GF mice fed a Malawi-style diet; these mice exhibited malnutrition and reduced weight gain compared with mice transplanted with the stool of healthy controls [36]. Indeed, Mack et al., among others, found altered gut microbiota in patients with AN. Furthermore, these authors showed significant differences between the gut microbiota of the restrictive and binge-purging subtypes of patients with AN [14]. If these differences were potentially linked to different energy-extracting capabilities, it could help to explain the observation that restrictive subtype patients need many more calories to gain weight than their binge-purge subtype counterparts [37]. The abundance of *Lactobacillus reuteri* was found to be positively associated with body mass index, while *Methanobrevibacter smithii* and *Bifidobacterium animalis* were negatively associated with body mass index in normal, overweight, and underweight patients [15]. Respective alterations in patients with acute AN partially normalized again upon weight restoration [13,14,15]. Certain *Lactobacillus* species are linked to obesity, diabetes and weight gain [15], while *M. smithii* are linked to energy exploitation, as it is capable of exploiting excessive H_2_ in the gut and metabolizing it into methane, increasing the transformation of nutrients into calories [10]. Finally, Hata et al. recently presented the first study of the transplantation of stool from patients with AN into GF mice [20]. As growth and development without gut bacteria has serious detrimental implications for immunological and brain development, these authors investigated the offspring of these transplanted GF mice. The offspring indeed showed reduced appetite, lower weight gain and less energy use. Furthermore, these offspring exhibited obsessive-compulsive (marble burying) and anxious (open field) behaviors that are often also observed in patients with AN, exemplifying the gut–brain interaction in these animals. Thus, at least the maintenance of AN symptoms, such as difficulty in gaining weight and anxious and obsessive behaviors, appears to be influenced by the gut microbiota, potentially increasing the risk of chronicity. One can speculate that at the beginning of the disease, a role of the gut microbiota appears conceivable. On the one hand, there is the important genetic basis of AN as mentioned above. On the other hand, we also know of a (limited) genetic component of each personal gut microbiota. Genetic influences may act via certain innate immune cell interactions or via glycoproteins that are expressed by gut cells. In the latter case, enterocytic surface-glycoproteins reach into the gut lumen and allow specific gut bacteria to better attach to the gut wall than others [38,39]. The genes of patients with AN could thus in part predispose them to certain gut bacteria favoring weight loss when dieting. These potential pathomechanisms, however, need to be further researched, as not enough data are available to date.

## 3. Altered Intestinal Permeability and Inflammation

The microbiota also plays an important role in modulating intestinal permeability via cells of the gut wall and regulation of intermediary tight junctions [40]. While the underlying mechanisms are not yet completely understood, they include bacterial products, such as lipopolysaccharides (LPS) [41] and short-chain fatty acids [42] as well as micro-RNA [43] that interact with gut wall enterocytes and tight junctions, e.g., via AMP-activated protein kinases (short-chain fatty acids) or cell differentiation, migration and architecture (micro-RNA). Stress and increased cortisol levels, as well as excessive exercise, have been found to increase this permeability [44]; all three are also typical for patients with AN. Some researchers have indeed found an increase in gut permeability in AN; however, recently, there have also been conflicting findings. Monteleone et al. [45] found reduced permeability in patients with AN when studying the small intestine using mannitol and lactulose absorption, and Mörkl et al. [46] did not find any difference using zonulin levels in the blood. On the other hand, Jesus et al. [47] showed that compared to normally fed mice, mice in the activity-based anorexia (ABA) animal model had reduced wall thickness, fewer tight junction proteins and increased permeability in the colon but not in the small intestine. ABA is the most widely used animal model of AN, and it combines reduced food with access to a running wheel, leading to paradoxically increased running wheel activity despite dramatic weight loss [48]. Achamrah et al. [49] could support the latter results with fluorescently labeled dextran in the ABA model in the colon. Furthermore, the gut microbiota in patients with AN appear to be shifted towards protein- and mucin-degrading Verrucomicrobia and Firmicutes with reduced Bacteroidetes, which mainly feed on carbohydrates [13,14]. This shift could be influenced by the reduced consumption of fat and carbohydrates in patients with AN and by the general reduction in their caloric intake and state of semistarvation. Mucin degraders have an evolutionary advantage over other bacteria under this condition, as they can also feed on the mucins produced by intestinal goblet cells that line the inside of the gut wall. A missing protective mucin layer might, however, further weaken the intestinal barrier and increase gut permeability [14,47]. Additionally, LPS released from increasingly starved gram-positive bacteria that depend on carbohydrate processing could cause an inflammatory reaction (see below) and further increase gut wall permeability. In summary, when pure abundance is examined, there is evidence in the AN animal model of increased permeability in the colon, where most bacteria are located, while the conditions in the small intestine are less clear. However, more multimodal research using functional tests for colonic permeability in patients and more precise insight into the differentiating underlying mechanisms using animal models are needed. Semistarvation-driven selection processes of bacterial taxa could further increase gut permeability.

A weakened barrier and increased permeability in the gut have far reaching consequences for the inflammatory and immunological reactions of the host. A greater passage of bacteria and bacterial parts, such as LPS or other bacterial products, into the cells of the gut wall, interstitial spaces and bloodstream can activate immune cells, increase proinflammatory cytokines and initiate antibody formation [22,50,51]. Two meta-analyses comparing patients with AN and healthy controls indeed showed low-grade inflammatory states, with increased TNF-alpha, IL-6 and IL1-beta production [52,53]. A greater role for inflammation in AN than previously thought is thus suspected, with chronic oxidative stress as well as physiological/psychological stress and abnormal bone marrow microenvironment additionally contributing to potential immune dysregulation [54]. Furthermore, Fetissov’s group showed that bacterial components, e.g., of certain *E. coli*, when fed to mice, evidently translocate via the gut wall and induce antibody production [55]. In some cases, these antibodies can act as partial homologues to neuropeptides and cross-react with receptors of appetite- and satiety-regulating hormones, such as alpha melanocyte-stimulating hormone and ghrelin, subsequently altering feeding behavior [56,57,58]. Even more interestingly, these antibodies were also shown to be elevated in patients with AN and bulimia nervosa and correlated with eating disorder psychopathology [58,59,60]. Autoantibodies due to increased gut permeability could thus play an important new role in the pathophysiology of AN and help to explain reduced hunger, increased satiety, and reduced hedonistic reward by food, as commonly reported by patients with AN [30,56]. Finally, patients with AN showed an interesting increase in autoimmune diseases; in particular, patients with AN exhibit an approximately two-fold increase in gastrointestinal and endocrine autoimmune diseases, including type 1 diabetes, and even a four-fold increase in Crohn’s disease [61,62]. Conversely, patients with autoimmune diseases also more commonly reported eating disorders and AN [63]. This elevated autoimmunity could potentially also be explained by increased gut permeability, translocating bacterial components and the ensuing immunological response to partially homologous bacteria. Other mechanisms of this bidirectional relationship might include overlapping genes, as found in the first genome-wide significant association in AN identified in a region previously implicated in autoimmune diseases [64]. Furthermore, colitis and Crohn’s disease patients often report eating behavior changes, as they might perceive certain foods as risk factors for relapse and thus reduce their consumption or find them less pleasurable [65]. Immune system alterations, chronic inflammation and increased stress hormone levels are also shared by inflammatory bowel diseases and AN and may be involved as mediating mechanisms between the two [61]. 

## 4. First Findings in Patients with AN and Animal Models

Six cross-sectional and three longitudinal studies with a total of 187 patients have been published to date on the gut microbiota of patients with AN [10,11,12,13,14,15,16,17,66], with varying results [28,30,67]. Several studies found a reduction in bacterial diversity in patients with acute AN [13,68], but others did not [11,14,69]. Reduced bacterial diversity can be a sign of dysbiosis, defined as a “microbial imbalance or maladaptation on or inside the body” [70,71]. In this case, certain species and their functions are lost to the bacterial community, and this is often associated with a reduced capacity to react to specific environmental changes or pathogens. Diseases with reduced gut bacterial diversity include posttraumatic stress disorder, obesity and inflammatory bowel diseases; in the last case, reduced diversity is correlated with increased intestinal permeability and inflammation [72]. In AN, some authors have linked reduced bacterial diversity to more severe eating disorder symptoms as well as comorbid depression [13]; however, the findings have not been replicated thus far. Following weight rehabilitation, several authors have found an increase in diversity [13,14]. Interestingly, patients in Mack et al.’s study and in our own study showed an increased absolute level of diversity after short-term weight recovery compared to healthy controls [14,69]. This puzzling result could point to short-term adaptive processes; be a result of the selection of generally more “healthy” foods, including large amounts of fiber, fresh fruits and vegetables and fewer highly processed foods, by patients with AN; or signify the increase in maladaptive species due to, for example, still unbalanced food selection, high stress levels or not-yet-normalized hormonal status, even after short-term weight recovery [3]. Longer follow-up studies are needed to understand the long-term gut microbiota diversity in completely recovered patients with AN.

When looking at which species are present within this diversity, overall beta-diversity measures can be calculated to evaluate the general similarity between two bacterial communities, or the abundance of specific taxa can be directly compared. Regarding the first measure, studies have found globally altered bacterial communities in patients with acute AN [13,14], which were not normalized with short-term weight recovery in either adults or adolescents [14,69], emphasizing again that the microbiota composition shifts in AN are more than an epiphenomenon of low weight. Regarding single taxa analyses, the results varied widely, and some of the more commonly found differences include increased Firmicutes and decreased Bacteroidetes abundances at the phylum level [13,14]. Furthermore, *Roseburia* and *Lactobacillus* species decreased in adults with increases in *Akkermansia* and the archaea *Methanobrevibacter smithii* [10,11,14,15], while *Romboutsia* and Enterobacteriaceae species decreased, Lachnospiracea species increased in the first study with exclusively adolescent patients [69]. This highlights potential age- and developmentally dependent effects that might be due to different hormonal states and habits, such as exercise or sleep, in adolescents or a generally shorter illness duration in younger patients. *Methanobrevibacter smithii* has been associated with increased energy exploitation [14], and its presence was inversely correlated with body mass index in one study [15]. *Roseburia* feed on carbohydrates, which are heavily reduced in the average diet of patients with acute AN; *Roseburia* can metabolize these carbohydrates to produce butyrate, which is known to help protect the gut wall barrier and decrease permeability. A decrease in *Roseburia*, together with increases in mucin-degrading Firmicutes and *Akkermansia*, could thus lead to further increases in gut permeability [73] and inflammation [74]. Indeed, one study analyzing protein degradation products showed reduced unbranched-chain fatty acids (such as butyrate), while two studies showed increased levels of branched-chain fatty acids that led to increased PYY production, which is linked to increased depression and reduced appetite [14,75].

Interestingly, in our study in adolescents, species belonging to the Lachnospiraceae family helped to predict short-term clinical outcome in the form of shorter treatment duration, independent of other known predictors, such as illness duration and low body weight at admission [69]. Lachnospiraceae are associated with weight gain following antibiotic treatment [76] and increased blood glucose levels [77] and could thus be linked to energy use and more rapid weight gain. Lachnospiracea also produces butyrate [78], thus affecting the intestinal barrier. A Lachnospiracea reduction is also the hallmark of gut inflammatory diseases, such as Crohn’s disease, and systemic autoimmune diseases, such as systemic lupus and multiple sclerosis [73,79]. As all these diseases are also characterized by microbial dysbiosis, another mechanism of Lachnospiracea could be immune and inflammatory processes [80]. By administering butyrate-producing bacteria, Geirnaert et al. could effectively modulate Crohn’s disease patients’ butyrate levels and increase gut wall integrity [73]. Therefore, further elucidating the function of these species in AN might be an interesting option for future treatment research. Oral treatment with gut bacteria, however, is not an easy task, as they have to be extracted, cultured and encapsulated, sometimes under anaerobic conditions; once these steps have been achieved, oral capsules can present a safe and successful route of administration, as shown for fecal transplantations in patients with *Clostridium difficile* infections [81].

To date, two case studies have been reported on transplanting stool from healthy donors into patients with AN. While the first study resulted in a 6-kg weight increase in a patient ill with AN for 2 years [19], the second study failed to help a chronically ill patient gain weight [18]. Notably, de Clerq et al. showed an increase in *Akkermansia muciniphila* species and a decrease in resting state energy consumption after transplantation, which lasted until at least the 36-week follow-up period [19]. Prochazkova et al. showed that microbial markers and dysbiosis improved, e.g., both diversity and total short-chain fatty acids increased. However, the chronically ill patient in that study failed to comply with the advice of the nutritionist; thus, the lack of weight gain in this patient could be attributed purely to nutritional reasons [18].

Using the ABA animal model, Queipo-Ortuno et al. confirmed widespread changes in the gut microbiota in food-restricted animals, some of which, including *Lactobacillus* and *Bifidobacterium* species, were associated with serum leptin and inversely associated with serum ghrelin concentrations [82]. Leptin is known to induce satiety and is severely reduced in patients with AN, while the appetite-inducing gastric peptide ghrelin is increased [83]. Breton et al. were also able to show starvation-induced microbiota alterations in ABA mice [84]. Sixty-eight amplicon sequence variants were less common, and eight were more abundant, including several *Lactobacillus* species. Several of these bacteria were correlated with body weight as well as neuropeptide Y and pro-opiomelanocortin levels in the hypothalamus, which are potentially also involved in appetite regulation [84]. Regarding the gut–brain axis, Möhle et al., using antibiotics to eradicate gut bacteria, showed direct implications of a reduction in neuronal cell neogenesis in the hippocampus and a loss of learning capacity in mice. *Bifidobacteria* and *Lactobacillus* species, given as probiotics, were able to reverse these effects [85]. Our own group showed similar learning deficits in ABA animals [86] as well as astrocyte loss associated with brain volume reduction [87,88], and the latter is also commonly found in patients with AN [89] and linked to neuropsychological deficits and negative prognosis [90]. Recent ABA results from our group appear to associate the diversity of the microbiota in the gut with these reductions in brain volume and astrocyte markers with the abundance of *Odoribacter* and *Bifidobacterium.* Interestingly, *Lactobacillus* abundance was positively linked to relative protection from brain volume loss, making it an interesting target for potential probiotic interventions [91].

## 5. Outlook

AN indeed appears to be a helpful “model” for studying the microbiota–gut–brain interactions, resulting in several findings regarding weight gain, intestinal permeability, inflammation, antibody formation and associated eating disorder and comorbid psychopathologies. Systematically studying the gut microbiota in patients with AN and the ABA animal model, paying specific attention to causal effects, will help us to gradually unravel these complex interactions and gain a better understanding of the underlying pathophysiology. Screening the gut microbiota in acutely ill patients with AN might help us discern those most at risk. However, as specific findings appear to be heterogeneous, unified analysis pipelines between research sites are needed. Additionally, taxonomic designations of community structure, as reported in the first studies of AN, might not be sufficient to adequately understand the underlying mechanisms. Potentially, the metabolic capacities of the gut microbiota need to be studied in greater detail, e.g., using metagenomics, transcriptomics and metabolomics approaches, preferably in synchronous multiomics and longitudinal experiments to also expose their interactions [92,93]. For better predictive power, we also need larger studies with longer-term follow-up, e.g., predictive findings of Lachnospiraceae abundance for the clinical course. Well-controlled studies of supplementation with Lachnospiraceae, *Lactobacillus*, and other potentially helpful pre-, pro- and synbiotics in animal models and patients will help to elucidate the abovementioned causal effects and evaluate the possibility of microbial remediation therapy as an adjunct to classical weight rehabilitation and psychotherapy. Here, a recent systematic review on probiotic use in depression and anxiety found evidence for the effectiveness of these approaches despite limitations due to the application of different bacterial strains [94].

Nutritional supplements with a known influence on the gut microbiota, such as omega-3 fatty acids, are another promising research topic for the treatment of AN. These supplements have been shown to reduce local and systemic inflammation, potentially by reducing LPS-producing taxa, such as Enterobacteriaceae, via omega-3 fatty acid hydrolysis products in the lower intestine and increasing butyrate-producing taxa, such as the abovementioned Lachnospiraceae or *Akkermansia muciniphila*, leading to reduced gut wall permeability. These supplements can also help with weight gain and favorably influence brain development (for a review, see [95]). A first small study in patients with AN showed a trend towards increased energy use [96]. Several randomized controlled studies supplementing omega-3 fatty acids are currently underway [97,98].

However, standard refeeding therapies for patients with AN need to be critically evaluated and systematically researched. Refeeding is typically conducted with “normal” hospital food and sometimes involves oral cow milk-based supplements or nasogastric feeding formula. However, we know little about the direct and indirect effects of these foods on the gut microbiota and their interaction with the host [29,30]. For example, changing from a nonanimal-based to an animal-based diet alone already has a marked effect on the gut microbiota within 1–2 days [99]. This could be highly relevant, as the majority of patients with AN consume vegetarian or vegan food before being admitted to the hospital; thus, rapidly changing to an animal-based diet will probably have an effect on their gut microbiota [29,30]. Furthermore, physicians must take into account that 25% of the “regular”, nonantibiotic drugs also have a measurable effect on the gut microbiota [100], which should be monitored. As we gain more knowledge about food and drug interactions with the gut microbiota, we should also better tailor these nutritional treatments to patients’ individual needs. Thus, more systematic research on refeeding and nutrition, as well as on what foods should be selected, at what times, and in what specific quantities to achieve the specific desired effects on the gut microbiota, is urgently needed in patients with AN to optimize refeeding therapy.

As over 80% of patients with AN also show gastrointestinal symptoms, ranging from constipation, bloating and distension to outright pain following food consumption [14], a better understanding of the role of gut microbiota–host interactions might also help to alleviate some of these problems. These patients suffer, and their symptoms also often cause patients to eat less, thus making refeeding more difficult and possibly playing a role in the development of chronicity [101]. Interestingly, in a large register-based epidemiologic study with over 15,000 patients with eating disorders, patients had a 2.5-fold increased chance of receiving a gastrointestinal diagnosis and a prescription of gastrointestinal drugs even in the two years prior to the onset of the eating disorder [102]. However, gastrointestinal symptoms and their eating-associated discomfort might also play a role in the development of AN.

Taken together, studying microbiota gut–brain interactions and using nutritional interventions and supplementation appear to hold great promise for further elucidating the underlying pathophysiological mechanisms that contribute to AN as well as for developing additional treatment options against this chronic and crippling disease.
